# Prevalence and risk profile of cervical human papillomavirus infection in Zhejiang Province, southeast China: a population-based study

**DOI:** 10.1186/1743-422X-7-66

**Published:** 2010-03-23

**Authors:** Jing Ye, Xiaodong Cheng, Xiaojing Chen, Feng Ye, Weiguo Lü, Xing Xie

**Affiliations:** 1 Women's Reproductive Health Key Laboratory of Zhejiang Province, Women's Hospital, School of Medicine, Zhejiang University, Xueshi Rd#2, Hangzhou, 310006, PR China; 2 Women's Reproductive Health Key Laboratory of Zhejiang Province; Department of Gynecologic Oncology, Women's Hospital, School of Medicine, Zhejiang University, Xueshi Rd#2, Hangzhou, 310006, PR China

## Abstract

**Background:**

Benign or malignant lesions caused by Human papillomavirus (HPV) produce considerable health burden worldwide. Since limited cross-protection would be offered between HPV types, heterogeneity in HPV type-specific distribution should be taken into account when predicting the effect of current prophylactic vaccines and forming the basic for the second-generation vaccines targeted to specific regions. Here, we investigated the prevalence of cervical HPV infection, as well as predictors concerned, in Zhejiang Province, southeast China.

**Results:**

Totally 4987 cervical samples from five randomly chosen counties in Zhejiang Province were detected. The overall HPV prevalence was 13.3%. Established high-risk (HR) HPV prevalence was 10.2%. HPV-52 was the most prevalent type (3.1%), followed by HPV-16 (2.5%), -58 (2.1%), -68 (1.0%) and -81 (0.9%). HPV-16 or -18 were present in 3.1% of the detected samples, while 174 specimens (3.5%) were positive for any of HPV-6, -11, -16 and -18. The prevalence of HPV vaccine types was consistently low across age groups. Bimodal age distribution in HR-HPV, including established HR and probably HR-HPV, was observed, with a clear second peak in perimenopausal women. Multivariate unconditional logistic regression models revealed that partner's lifetime number of partners was the only common independent predictor of overall, established HR, established low-risk, single-type and multiple-type HPV infection in current study.

**Conclusions:**

We have observed low prevalence of HPV vaccine types and relatively high prevalence of HPV-52 and -58 in our population. Our findings support universal "catch-up" vaccination of sexual experienced young women in Zhejiang Province, as well as enhance the hypothesis that the second-generation HPV prophylactic vaccines including HPV-52 and -58 may offer higher protection for women in China and other Asian areas. Furthermore, our data support close surveillance of perimenopausal women with HR-HPV infection.

## Background

Papillomaviruses (PVs) comprise of a large group of non-enveloped double-stranded DNA viruses that infect a variety of vertebrate species, including humans. There are 16 genera of PVs and five of these genera, alpha, beta, gamma, mu and nu, are human papillomaviruses (HPVs). HPV infection may exist asymptomatically or induce the formation of benign or malignant lesions of human mucosal and cutaneous epithelia. HPV has been shown to be the etiologic agent of anogenital, especially cervical, cancers and a fraction of oropharyngeal carcinomas. Other cancers causally linked to HPV include non-melanoma skin cancer and conjunctiva carcinoma [1,2]. More than 100 HPV genotypes have been molecularly characterized [1] and the types associated with anogenital cancers, which are of greatest medical importance, belong to the alpha-PV genus. According to International Agency for Research on Cancer working group, HPV-16, -18, -31, -33, -35, -39, -45, -51, -52, -56, -58, and -59 were classified as "carcinogenic to humans", while HPV-6 and -11 were classified as "not classifiable as to its carcinogenicity to humans" [3].

Both the two available prophylactic HPV vaccines, the bivalent vaccine (Cervarix^®^, GlaxoSmithKline Biologicals, targeted at HPV-16 and -18) and the quadrivalent vaccine (Gardasil^®^, Merck, targeted at HPV-6, -11, -16 and -18), have shown prominently type-restricted prophylactic efficacy for genital lesions related to targeted types in women who were naive to the respective types at enrollment [4-7]. HPV vaccination also promises to decrease the incidence of other HPV-related cancers, as well as reduce the burden associated with the treatment of HPV-related benign lesions [8]. Since limited cross-protection would be offered between HPV types [9], heterogeneity in HPV type-specific distribution from different populations should be taken into account when predicting the effect of current prophylactic vaccines [10] and forming the basic for the second-generation vaccines targeted to specific regions.

Since March 2009, a randomized, double-blind trial testing the safety and efficacy of the quadrivalent vaccine (Gardasil^®^, Merck) in Chinese women (V501-041-00) was conducted in Zhejiang Province, a coastal region in southeast China. To the best of our knowledge, no epidemiologic data on HPV genotypes in general female population were reported from Zhejiang Province, which highlights the need for timely population-based study in this region. Due to the feasible anatomic site for sampling and the well-established sampling method for cervix, here, we investigated the cervical HPV infection in asymptomatic general female population to be conscious of the overall, type-specific and age-specific HPV prevalence, as well as determinants of HPV infection, in Zhejiang Province before large scale vaccination programs occur.

## Methods

### Study population and inclusion criteria

A total of 7500 women were randomly selected as candidates between November 2007 and August 2008 from the population list of five randomly chosen counties. These five counties are located in east, south, west, north and middle part of Zhejiang Province, respectively. In each county, one town and one village were randomly selected, and 1500 women (750 town and 750 village residents, respectively) were randomly recruited to ensure geographical and social diversity. A woman was eligible to be study objective if she: (a) was mentally and physically competent; (b) was aged between 20 and 79 years; (c) was a permanent resident of local area; (d) had no history of abnormal cytology or cervico-vaginal dysplasia; (e) had no abnormal vaginal bleeding and contact bleeding; (f) had no visual cervical lesions at simple visual inspection during gynecological examination; (g) had no history and associated symptoms of other HPV-related diseases; (h) was not a virgin; (i) was not presently pregnant; (j) had not undergone a total hysterectomy; (k) had no use of vaginal medication and no sexual intercourse in the previous three days. Eligible women were invited for a face-to-face interview and a gynecological examination in local hospitals.

Of these 7500 enumerated women, 247 stayed away from home at interview time, 376 did not meet the inclusion criteria, 317 could not undergo gynecological examination because of menstruation, and 1502 refused to participate mainly because they did not have enough time or did not think they needed gynecological examination in the absence of symptoms. Totally 5058 women were enrolled into the study.

All participants agreed to participate in the present study and signed informed consent forms. The study project was approved by The Human Research Ethical Committee of Women's Hospital, School of Medicine, Zhejiang University.

### Questionnaire interview

An interviewer-administrated structured questionnaire was designed to collect information on socio-demographic characteristics, lifestyle, menstrual status, reproductive history, contraception and sexual behavior. Trained research nurses questioned participants face-to-face in the local dialect of Chinese.

### Cervical specimen collection

Samples of exfoliated cervical cells were collected with DNAPap cervical sampler (Digene) during gynecological examinations. The sampler was inserted 1-1.5 cm into the endocervical canal and rotated 3-5 full turns in counterclockwise direction. The tip containing cellular material was then placed into transport medium tube and stored at 4°C immediately. All specimens were coded without knowledge of the subjects.

### HPV detection

HPV DNA was extracted with phenol-chloroform, resuspended in 100 μL elution buffer, and stored at -30°C. The quality and integrity of sample DNA for polymerase chain reaction (PCR) was verified by amplification of a 268 base pair region of the human β-globin gene [11]. Specimens with negative internal control amplification were excluded. All the β-globin-positive samples were amplified with the MY09/11 L1 consensus primers of anogenital HPV genotypes [12] and synchronously detected with the commercially introduced HPV GenoArray Test kit (Hybribio). All the MY09/11 consensus PCR-positive/GenoArray-negative samples were typed with an improved PCR-restriction fragment length polymorphism (RFLP) assay validated by Hybrid Capture II and sequencing [13]. Approximately 7.5% MY09/11 consensus PCR-positive/GenoArray-positive samples and 10% MY09/11 consensus PCR-RFLP typed samples were randomly selected to be validated by sequencing. More than 5% MY09/11 consensus PCR-negative/GenoArray-negative samples were randomly selected to be validated by Real Time PCR HPV Detection Kit (Hybribio).

#### PCR

PCR was performed in 50 μL reaction mixture containing 10× PCR buffer, 25 mM MgCl_2_, 200 μM deoxynucleotide triphosphate, 2 U Taq polymerase, 10 pmol of each primer, and 5 μL template DNA. The PCR protocol was: preheating at 94°C for 7 min, followed by 36 cycles of denaturation at 94°C for 45 sec, annealing at 56°C for 45 sec, and extension at 72°C for 45 sec, at last a final extension at 72°C for 10 min. Products were identified by electrophoresis in 2.0% agarose gel stained with ethidium bromide. For every PCR assay, a negative control and a positive control (HPV DNA from SiHa) were run to control for possible contamination and accuracy.

#### HPV GenoArray Test

HPV GenoArray Test kit makes use of both DNA amplification and Hybribio's proprietary Flow-through Hybridization Technology to simultaneously identify 21 HPV genotypes: HPV-6, -11, -16, -18, -31, -33, -35, -39, -42, -43, -44, -45, -51, -52, -53, -56, -58, -59, -66, -68 and -81 (equivalent to CP8304). The test employs a gene chip with a nylon membrane onto which type-specific oligonucleotides probes have been immobilized. The final results were detected by colorimetric change on the chip under direct visualization. Test was performed according to the manufacture's instructions.

#### Genotyping by RFLP

The products generated by MY09/11 L1 consensus primers were typed with RFLP as described by Hong *et al*. [13].

#### Sequencing

MY09/11 consensus PCR-amplified products were sequenced in the 3730 ABI Prism sequencer (Applied Biosystems). The obtained sequences were compared to known HPV sequence databases (available at: http://www.ncbi.nlm.nih.gov/BLAST/).

#### Real Time PCR HPV Detection

Real Time PCR HPV Detection kit detects 13 HPV types (HPV-16, -18, -31, -33, -35, -39, -45, -51, -52, -56, -58, -59 and -68) in one single reaction by real-time PCR equipment. Test was performed according to the manufacture's instructions.

### Definition and estimation

Positive result by any assay mentioned above was regarded as HPV positive. For samples inconsistent between HPV GenoArray Test and sequencing, the genotypes were identified according to sequencing. Multiple-type infection was separated into constituent types, thus type-specific prevalence included that in both single-type and multiple-type infection. Based on epidemiologic classification associated with cervical cancer, HPV-16, -18, -31, -33, -35, -39, -45, -51, -52, -56, -58, and -59 were classified as established high-risk (HR) types, HPV-26, -53, -66, -68, -73 and -82 were classified as probably HR types, and HPV-6, -11, -40, -42, -43, -44, -54, -61, -70, -72, -81 and -89 (equivalent to CP6108) were classified as established low-risk (LR) types [1,3]. All other HPV types were considered as undetermined risk HPV types. Analyses were repeated by grouping HPV types according to phylogeny [1,14].

### Statistical analysis

Data were key-entered twice and analyzed using SPSS software for Windows (version 16.0). The χ^2 ^test and, where appropriate, Fisher's exact test were used to compare HPV prevalence across age groups. Odds ratios (ORs) with 95% confidence intervals (CIs) were calculated using unconditional logistic regression to estimate independent predictors for HPV infection. Analyses were performed for overall, established HR, established LR, single-type and multiple-type HPV infection, respectively. Age group and variables associated with HPV positivity in age-adjusted model were included as candidates in multivariate model. Final model was determined by forward elimination of candidate variables based on likelihood ratio tests. In all of the logistic models, HPV-negative women were used as the reference group. All hypotheses testing were two-sided. A level of 0.05 was chosen to indicate statistical significance.

## Results

### HPV genotyping algorithm

**Figure 1:**
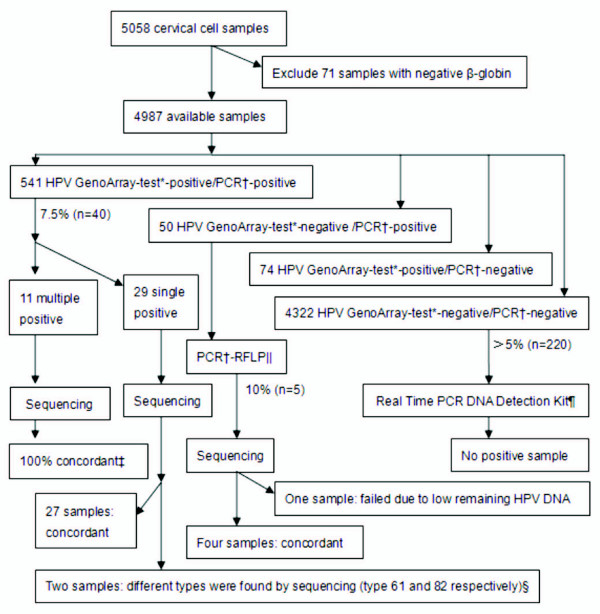
**HPV genotyping algorithm**. *HPV GenoArray-test kit detects 21 HPV genotypes: HPV-6, -11, -16, -18, -31, -33, -35, -39, -42, -43, -44, -45, -51, -52, -53, -56, -58, -59, -66, -68 and -81. † Refer to the MY09/11 consensus PCR. ‡ If the sequencing genotype was included in the result of HPV GenoArray-test, it was considered as concordance. §HPV-61 and -82 were not included in the HPV GenoArray-test gene chip. || The genotypes identified by MY09/11 consensus PCR-RFLP assay included HPV-13, -26, -40, -54, -57, -61, -62, -64, -82, -83, -85, -86, -87 and -89, all of which were not contained in the HPV GenoArray-test gene chip. ¶Real Time PCR HPV Detection kit detects 13 HPV types: HPV-16, -18, -31, -33, -35, -39, -45, -51, -52, -56, -58, -59 and -68.

Of 5058 women who provided cervical cell samples, 71 were excluded because of negative β-globin. In the leaving 4987 samples, 615 (12.3%) were HPV GenoArray-positive and 591 (11.9%) were MY09/11 consensus PCR-positive. There was a 97.5% overall agreement between these two assays (kappa = 0.883; *P *< 0.001). Figure 1 shows the detailed HPV genotyping algorithm.

### Epidemiological distribution of HPV types

In 4987 detected cervical samples, 665 (13.3%) were HPV positive. The corresponding prevalence, age-standardized to the world population, were 13.5% overall. Of these 665 positive samples, 508 (76.4%) were established HR-HPV and 176 (26.5%) were multiple-type HPV infections. Totally 35 different genotypes, grouped in 11 alpha species, were identified. The five most prevalent types were HPV-52 (3.1%), HPV-16 (2.5%), HPV-58 (2.1%), HPV-68 (1.0%) and HPV-81 (0.9%) (Table 1). Stratified with counties, the most prevalent types in east county were HPV-52 (37/1003, 3.7%), HPV-58 (27/1003, 2.7%), HPV-16 (17/1003, 1.7%), HPV-68 (9/1003, 0.9%), HPV-51 (7/1003, 0.7%) and HPV-53 (7/1003, 0.7%); in south county were HPV-52 (30/1045, 2.9%), HPV-16 (25/1045, 2.4%), HPV-68 (18/1045, 1.7%), HPV-58 (17/1045, 1.6%) and HPV-81 (16/1045, 1.5%); in west county were HPV-52 (31/1042, 3.0%), HPV-16 (25/1042, 2.4%), HPV-31 (14/1042, 1.3%), HPV-33 (12/1042, 1.2%) and HPV-58 (12/1042, 1.2%); in north county were HPV-58 (23/934, 2.5%), HPV-16 (20/934, 2.1%), HPV-52 (20/934, 2.1%), HPV-81 (10/934, 1.1%) and HPV-68 (9/934, 1.0%); in middle county were HPV-52 (37/963, 3.8%), HPV-16 (36/963, 3.7%), HPV-58 (27/963, 2.8%), HPV-51 (15/963, 1.6%) and HPV-81 (14/963, 1.5%). The four most commonly detected HPV species were alpha-9, -7, -6 and -3 (Table 1). HPV-16 or -18 were present in 3.1% of the detected samples, while only nine women (0.2%) were infected with both HPV-16 and -18. Totally 174 women (3.5%) were positive for any of the vaccine types-6, -11, -16 and -18, while no women were simultaneously positive for all these four types (Table 1).

**Table 1:** HPV prevalence for specific types and categories, both overall and by age group (n = 4987)*.

			Prevalence by age group, %							
			
Category, HPV type (alpha species)	No.	Overall prevalence, %	20-24 (n = 117)	25-29 (n = 526)	30-34 (n = 825)	35-39 (n = 1102)	40-44 (n = 1041)	45-49 (n = 704)	50-54 (n = 368)	55-79 (n = 304)
Any type	665	13.3	16.2	12.9	11.0	13.9	13.6	13.2	17.7	11.2
Established HR types										
Any established HR types	508	10.2	14.5	9.3	8.4	10.7	10.7	9.2	14.4	8.6
16 (alpha-9)	123	2.5	1.7	2.5	2.2	2.4	2.1	2.8	4.1	2.3
18 (alpha-7)	39	0.8	0.9	0.6	0.8	0.9	1.2	0.4	0.5	0.3
31 (alpha-9)	39	0.8	1.7	1.0	0.4	1.3	0.5	0.4	0.8	1.3
33 (alpha-9)	38	0.8	2.6	1.0	0.6	0.7	0.7	0.4	1.4	0.7
35 (alpha-9)	9	0.2	0.9	0.0	0.4	0.0	0.1	0.1	0.5	0.3
39 (alpha-7)	13	0.3	0.0	0.2	0.4	0.1	0.4	0.0	0.0	1.3
45 (alpha-7)	16	0.3	0.9	0.8	0.2	0.1	0.4	0.4	0.0	0.3
51 (alpha-5)	46	0.9	0.0	1.3	1.0	0.5	0.6	0.6	2.2	2.3
52 (alpha-9)	155	3.1	3.4	2.1	2.7	3.4	3.8	3.3	3.3	2.0
56 (alpha-6)	21	0.4	1.7	0.4	0.1	0.2	0.4	0.6	1.1	0.7
58 (alpha-9)	106	2.1	3.4	2.3	1.8	1.9	2.0	1.8	3.5	2.3
59 (alpha-7)	33	0.7	0.0	1.1	0.6	1.0	0.2	0.3	1.1	1.0
Probably HR types										
Any probably HR types	120	2.4	4.3	3.2	1.3	2.4	1.6	3.1	4.1	2.3
26 (alpha-5)	3	0.1	0.0	0.2	0.0	0.0	0.1	0.0	0.3	0.0
53 (alpha-6)	39	0.8	2.6	0.6	0.6	0.7	0.5	0.7	1.4	1.6
66 (alpha-6)	27	0.5	0.0	1.3	0.1	0.5	0.3	0.9	0.8	0.3
68 (alpha-7)	52	1.0	1.7	1.1	0.5	1.2	0.8	1.7	1.6	0.3
82 (alpha-5)	2	0.0	0.0	0.0	0.1	0.0	0.1	0.0	0.0	0.0
Established LR types										
Any established LR types	137	2.7	2.6	2.3	2.3	2.7	2.8	2.3	4.3	3.9
6 (alpha-10)	10	0.2	0.0	0.2	0.2	0.0	0.2	0.0	0.0	1.6
11 (alpha-10)	11	0.2	0.0	0.2	0.4	0.3	0.2	0.0	0.3	0.3
40 (alpha-8)	1	0.0	0.0	0.2	0.0	0.0	0.0	0.0	0.0	0.0
42 (alpha-1)	22	0.4	1.7	0.4	0.4	0.3	0.5	0.3	0.5	1.0
43 (alpha-8)	14	0.3	0.0	0.0	0.2	0.5	0.4	0.1	0.5	0.0
44 (alpha-10)	29	0.6	0.0	0.6	0.4	0.7	0.9	0.6	0.5	0.0
54 (alpha-13)	1	0.0	0.0	0.2	0.0	0.0	0.0	0.0	0.0	0.0
61 (alpha-3)	8	0.2	0.0	0.2	0.2	0.1	0.0	0.3	0.5	0.0
81 (alpha-3)	46	0.9	0.9	0.4	0.5	1.0	0.8	1.1	2.2	1.3
89 (alpha-3)	2	0.0	0.0	0.0	0.1	0.1	0.0	0.0	0.0	0.0
Undetermined types										
Any undetermined types	13	0.3	0.0	0.2	0.6	0.3	0.2	0.3	0.0	0.0
13 (alpha-10)	1	0.0	0.0	0.0	0.0	0.1	0.0	0.0	0.0	0.0
57 (alpha-4)	2	0.0	0.0	0.0	0.0	0.1	0.1	0.0	0.0	0.0
62 (alpha-3)	4	0.1	0.0	0.0	0.2	0.0	0.0	0.3	0.0	0.0
64 (alpha-11)	1	0.0	0.0	0.2	0.0	0.0	0.0	0.0	0.0	0.0
83 (alpha-3)	1	0.0	0.0	0.0	0.0	0.1	0.0	0.0	0.0	0.0
85 (alpha-7)	1	0.0	0.0	0.0	0.0	0.0	0.1	0.0	0.0	0.0
86 (alpha-3)	1	0.0	0.0	0.0	0.1	0.0	0.0	0.0	0.0	0.0
87 (alpha-3)	2	0.0	0.0	0.0	0.2	0.0	0.0	0.0	0.0	0.0
Vaccine types										
16 or 18	153	3.1	2.6	2.7	2.8	3.1	3.1	3.1	4.6	2.6
16+18	9	0.2	0.0	0.4	0.2	0.2	0.2	0.1	0.0	0.0
6 or 11 or 16 or 18	174	3.5	2.6	3.0	3.4	3.4	3.5	3.1	4.9	4.6
Single-type infections	489	9.8	12.0	9.3	7.9	10.7	10.7	10.4	10.9	6.2
Multiple-type infections	176	3.5	4.3	3.6	3.2	3.2	3.0	2.8	6.8	4.9
Alpha-1 type infections	22	0.4	1.7	0.4	0.4	0.3	0.5	0.3	0.5	1.0
Alpha-3 type infections	62	1.2	0.9	0.6	1.5	1.2	0.8	1.7	2.4	1.3
Alpha-4 type infections	2	0.0	0.0	0.0	0.0	0.1	0.1	0.0	0.0	0.0
Alpha-5 type infections	51	1.0	0.0	1.5	1.1	0.5	0.8	0.6	2.4	2.3
Alpha-6 type infections	81	1.6	3.4	2.3	0.8	1.4	1.1	2.0	3.0	2.3
Alpha-7 type infections	147	2.9	3.4	3.6	2.3	3.2	2.9	2.6	3.3	3.3
Alpha-8 type infections	15	0.3	0.0	0.2	0.2	0.5	0.4	0.1	0.5	0.0
Alpha-9 type infections	409	8.2	12.8	6.7	6.8	8.6	8.5	8.0	11.4	7.2
Alpha-10 type infections	51	1.0	0.0	1.0	1.0	1.1	1.2	0.6	0.8	2.0
Alpha-11 type infections	1	0.0	0.0	0.2	0.0	0.0	0.0	0.0	0.0	0.0
Alpha-13 type infections	1	0.0	0.0	0.2	0.0	0.0	0.0	0.0	0.0	0.0

HPV: human papillomavirus, No.: number, HR: high-risk, LR: low-risk

*The prevalence of an individual type or category included detection of this type or category in both single- and multiple-type infection.

**Figure 2:**
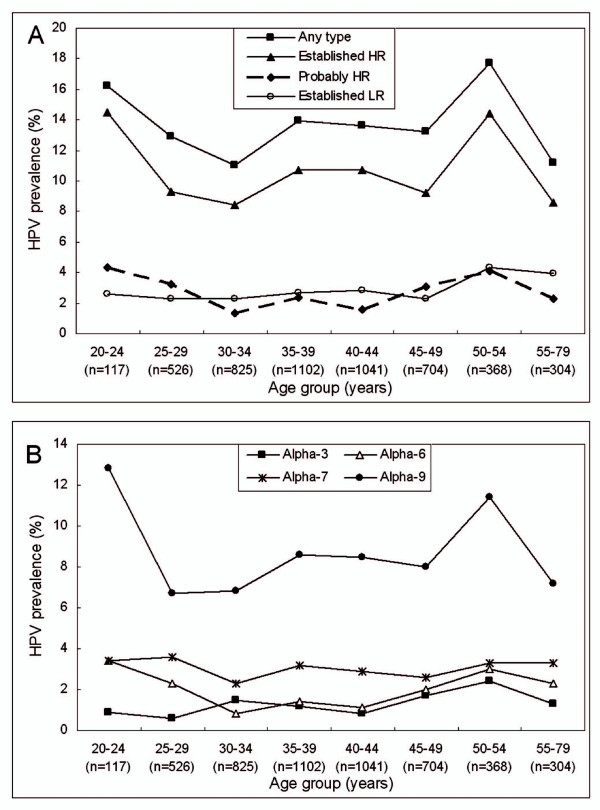
**Age-specific prevalence of HPV infection in general female population in Zhejiang Province, southeast China**. **(A) Overall HPV, established high-risk (HR) HPV, probably HR-HPV and established low-risk (LR) HPV**. Established HR-HPV types included HPV-16, -18, -31, -33, -35, -39, -45, -51, -52, -56, -58 and -59; probably HR-HPV types included HPV-26, -53, -66, -68, -73 and -82; established LR-HPV types included HPV-6, -11, -40, -42, -43, -44, -54, -61, -70, -72, -81 and -89 (equivalent to CP6108). **(B) Alpha-3, -6, -7 and -9 species**. Alpha-3 species included HPV-61, -62, -72, -81, -83, -84, -86, -87 and -89; alpha-6 species included HPV-30, -53, -56 and -66; alpha-7 species included HPV-18, -39, -45, -59, -68 -70 and -85; alpha-9 species included HPV-16, -31, -33, -35, -52, -58 and -67. The prevalence of an individual category included detection of this category in both single-type and multiple-type infection.

The mean age of the detected women was 39.7 (range 20-79, SD = 8.9) years. Figure 2 shows the age-specific prevalence for categories of HPV infection. There were two peaks of established HR-HPV infection. The first was 14.5% in women 20-24 years old and the second was 14.4% in women 50-54 years old (χ^2 ^= 15.166, *P *= 0.034) (Figure 1A). Similar pattern was observed for probably HR-HPV infection (χ^2 ^= 15.901, *P *= 0.026), while overall and established LR-HPV infection showed relatively flat curves (χ^2 ^= 12.284, *P *= 0.092 and χ^2 ^= 6.821, *P *= 0.448, respectively) (Figure 1A). The age-specific curves for alpha-9 (χ^2 ^= 12.998, *P *= 0.072) and -6 (χ^2 ^= 15.214, *P *= 0.033) were similar to the established HR and probably HR-HPV infection, with two peaks, while the curves for alpha-7 (χ^2 ^= 2.944, *P *= 0.890) and 3 (χ^2 ^= 9.898, *P *= 0.194) were more flat (Figure 1B). The prevalence for HPV-16 or-18 and for HPV-6, -11, -16 or -18 showed no significant differences across age groups.

### Risk profile for HPV Infection

In age-adjusted models, no significant associations were found between HPV positivity and smoking, income, age at menarche, age at marriage, reproductive history, condom or intrauterine device use as the main contraceptive method in the recent two years, history of malignancy, history of subtotal hysterectomy and history of Papanicolaou screening (data not shown). In multivariate models, marital status, residence, passive smoking, menstrual cycle, outcome of initial pregnancy, oral contraceptive use as the main contraceptive method in the recent two years, age at sexual initiation and lifetime number of sexual partners were eliminated from the final models. The independent predictors for overall, established HR, established LR, single-type and multiple-type HPV infection were listed in Table 2, respectively. Partner's lifetime number of partners was the only shared predictor for all categories of HPV infection (Table 2).

**Table 2:** Multivariate OR and 95% CI for predictors of different categories of HPV infection*.

		Multivariate OR (95%CI) for HPV infection‡				
		
Characteristics	No.†	Any-type HPV infection	Established HR- HPV infection	Established LR- HPV infection	Single-type HPV infection	Multiple-type HPV infection
Age (years)						
20-24	117		2.02(0.99-4.10)			0.85(0.30-2.41)
25-29	526		1.16(0.69-1.96)			0.71(0.35-1.42)
30-34	825		1.09(0.66-1.77)			0.62(0.32-1.19)
35-39	1102		1.48(0.94-2.34)			0.64(0.34-1.19)
40-44	1041		1.40(0.88-2.21)			0.61(0.32-1.15)
45-49	704		1.16(0.72-1.89)			0.57(0.29-1.14)
50-54	368		1.93(1.16-3.19)||			1.42(0.73-2.76)
55-79	304		Ref			Ref
*P *for trend			0.035			0.041
Occupation						
Farmer	644	1.24(0.92-1.67)	1.21(0.87-1.70)		1.39(0.99-1.95)	
White collar	1246	1.29(1.01-1.65)||	1.18(0.89-1.56)		1.43(1.08-1.90)||	
Businesswoman	422	1.28(0.91-1.80)	1.28(0.87-1.87)		1.53(1.05-2.24)||	
Unemployed	1209	1.74(1.38-2.20)#	1.82(1.40-2.38)#		1.88(1.43-2.47)#	
Blue collar labor	1388	Ref	Ref		Ref	
Education level						
Under middle school	1589			Ref		
Middle school	2812			0.61(0.41-0.92)||		
University or above	582			1.15(0.66-2.02)		
Residence of partner						
Urban	1623			1.61(1.08-2.41)||		
Rural	3343			Ref		
Ever use of OC						
Former or Current	900	1.28(1.04-1.58)||	1.46(1.16-1.84)¶			
Never	4087	Ref	Ref			
Partner's No. of partners§						
1	4736	Ref	Ref	Ref	Ref	Ref
≥2	170	2.72(1.89-3.90)#	2.42(1.59-3.69)#	3.16(1.65-6.04)¶	2.83(1.90-4.22)#	2.71(1.49-4.95)¶

OR: Odds ratio, CI: Confidence interval, HPV: human papillomavirus, No.: number, HR: high-risk, LR: low-risk, OC, oral contraceptive, Ref: reference *The reference group was HPV-negative women.

† Numbers did not always add up to total due to missing data.

‡ Age group and variables associated with HPV infection in respective age-adjusted model were included as candidates in respective multivariate model. Final modals were determined by forward elimination of candidate variables based on likelihood ratio tests.

§Refers to partner's lifetime number of partners.

|| P < 0.05.

¶P < 0.01.

#P <0.001.

## Discussion

To date, no type-specific HPV testing is approved by the Food and Drug Administration or is used widely. HPV GenoArray-test, which has already been used for quite a while in European and Asian countries, showed high level agreement (97.5%) with MY09/11 consensus PCR system. The concordance of HPV GenoArray-test with Amplicor HPV test [15] or Roche Linear Array [16] was also reported to be excellent. Considering that HPV GenoArray-test could not identify genotypes not included in gene chip, we utilized an improved PCR-RFLP technique for the MY09/11 consensus PCR-positive/GenoArray-negative samples.

Compared with similar studies in China, age-standardized HPV prevalence estimation in Zhejiang Province (13.5%) was similar to that in a central rural area of China (14.2%) [17], but lower than that in the largest city in northeast China (16.6%) [18] and an urban city in south China (17.6%) [19]. Although 1502 women refused to participate, it may not cause large bias as HPV infection is asymptomatic. HPV prevalence in women without cervical abnormalities was estimated to between 1.4% to 25.6% by country, and varied from 1.6% to 14.2% in Asia countries [10]. The population in our study was asymptomatic women with no history of cervical neoplasia or other HPV-related diseases. Based on cost constraints, we did not perform the Bethesda System Terminology based cytology for participants in this study. On the one hand, it may actually help better estimate HPV burden in the general female population in this region. On the other hand, inclusion of women with abnormal cytology (mainly mild ones) did not materially affect significant findings [10,19-21], which made our findings comparable with other investigations.

The five most common HPV types in general female population worldwide were HPV-16, -18, -31, -58 and -52, while the rank varied by region [22]. Contrasting to most previous surveys in China [17-19] and other populations [22], HPV-52, rather than HPV-16, was the most commonly identified type in our population, consistent with the data from Japan and Taiwan and eastern Africa [22]. HPV-16 ranked the second and the prevalence (2.5%) was comparable with that worldwide [22]. HPV-58, another common type in Asian population [23,24], ranked the third (15.9% of all infections), similar to that in South Taiwan (19.9% of all infections in general population) [24]. The finding that HPV-52 was more prevalent than HPV-16 may be due to the geographical and biological interplay between HPV types or variants and host immunogenic factors [25]. Another interpretation attributed to the genotyping system. On the one hand, with HPV GenoArray-test kit, Lin *et al*. [26] found that HPV-52 and -58 were the most prevalent genotypes in Chinese women in Guangdong Province, while Grisaru *et al*. [15] found the relatively high percentage of HPV-52 in Israeli Jewish women referred for colposcopic examination. On the other hand, Liu *et al*. [16] reported that HPV GenoArray-test had an analytical sensitivity in detection of HPV-16 and -18 in as few as 10-50 copies. Larger and comprehensive studies are warranted to further evaluate the performance of HPV GenoArray-test, however, the preponderance of HPV-52 and -58 in our study population is meaningful. As accumulated studies had shown that HPV-52 and -58 were more predominant in Asian populations [10,17-19,27] and overrepresented in cervical cancer cases from eastern and southeastern Asia [13,28,29], we assume that the second-generation HPV prophylactic vaccines including HPV-52 and -58 may offer higher protection for women in China and other Asian areas.

For the current available prophylactic vaccines, the prevalence of vaccine types was consistently low across age groups. Since a proportion of older women might have already been infected and cleared previous infections, the actually exposed rate to vaccine types might be higher than observed in older women. Considering that HPV prophylactic vaccines do not have clinical benefit in women who have been infected with vaccine types at the time of vaccination, we assume that younger women would draw more benefit from "catch-up" HPV vaccination programs.

Bimodal age distribution of HR-HPV, which was not common in mainland China [17,18,26], was observed in the current study. Viral prevalence is the product of incidence, persistence and clearance of infection. As HPV is often acquired soon after sexual initiation [30], the actual first peak of HR-HPV infection might before 20 years old, due to the higher probability of exposure to new HPV infections and the lack of adaptive immune responses in younger women. The second peak observed in perimenopausal women may be partly explained by viral persistence or reactivation of latent HPV due to the physiologic and immunologic dysregulation caused by hormone fluctuations at menopausal transition [31]. Another interpretation attributes to changes in the sexual behavior of women or their partners in middle age. Considering that only HR-HPV, which had a higher tendency to cause persistent infection [32,33], showed the second peak around menopause, we assume that persistence or reactivation of latent HPV may better explain the second peak in our population. Castle *et al*. also suggested a stronger role for viral persistence than for acquisition of new infections in women aged 45 years or older in Costa Rica [34]. Accordingly, HR-HPV positivity around the age of menopause implies more possibility of viral persistence, which makes HPV screening have more important clinical meaning for perimenopausal women than for young women. For perimenopausal women with positive HR-HPV, further evaluation, including cytology and even colposcopy, should be considered and regular screening should be prolonged because they have higher risk for the development of cervical cancer.

Alpha-6, -7, -9 species comprised mainly of established HR and probably HR-HPV types, while alpha-3 species comprised mainly of established LR-HPV types. However, alpha-7 species were more closely genetically related to alpha-3 than to alpha-9 species [35]. Consistently, the age-related curves for alpha-7 and alpha-3 species were similar. The difference of age curves between alpha-3/7 species and alpha-6/9 species may be a reflection for different tropisms for cervical columnar epithelium. According to Castle *et al*., the age-related changes in cervix influence HPV type-specific detection at the cervical os, where cervical specimens are routinely collected [35]. What is more, diverse patterns also exist among different populations with regard to the same species [21,35]. Whether it is due to the diversity in the physiologic change of cervix among different populations, or it could be explained by variants which may have subtle differences in tropism and other biological behaviors, is unclear.

We also explored the independent predictors for categories of HPV positivity. Previous studies showed that high-risk sexual behavior, including early sexual initiation, multiple sexual partners and sexual partner as an HPV carrier, was the pivotal determinant for HPV infection among women [36-38]. However, less independent indicators related with sexual behavior was found in our study, except for partner's lifetime number of partners, which might be due to traditional habitude, especially in oriental countries. Furthermore, our finding suggest that in areas where women's sexual conception and behavior are conservative, men possessing multiple sexual partners may be a predominant resource for HPV positivity in women as long as HPV is mainly transmitted through sexual intercourse [39].

## Conclusions

This study provides a unique opportunity to gather baseline data on cervical HPV prevalence, as well as predictors concerned, in general female population in Zhejiang Province, southeast China. The data showed low prevalence of HPV vaccine types and relatively high prevalence of HPV-52 and -58. Our findings support universal "catch-up" vaccination of sexual experienced young women in this region, as well as enhance the hypothesis that the second-generation HPV prophylactic vaccines including HPV-52 and -58 may offer higher protection for women in China and other Asian areas. Furthermore, our data also support close surveillance of perimenopausal women with HR-HPV infection.

## Abbreviations

PV: papillomavirus; HPV: human papillomavirus; HR: high-risk; LR: low-risk; PCR: polymerase chain reaction; RFLP: restriction fragment length polymorphism; OR: odds ratio; CI: confidence interval.

## Competing interests

The authors declare that they have no competing interests.

## Authors' contributions

JY carried out the study, performed analysis of data and drafted the manuscript. XDC participated in the study design and coordination. XJC carried out the MY09/11 consensus PCR and the PCR-RFLP assays. FY contributed to study design and developed the assay protocol. WGL participated in the study design, sample collection and coordination. XX conceived the study, provided consultation and revised the manuscript. All authors have read and approved the final manuscript.
